# A Proof-of-Concept for Semantically Interoperable Federation of IoT Experimentation Facilities

**DOI:** 10.3390/s16071006

**Published:** 2016-06-29

**Authors:** Jorge Lanza, Luis Sanchez, David Gomez, Tarek Elsaleh, Ronald Steinke, Flavio Cirillo

**Affiliations:** 1 Network Planning and Mobile Communications Lab. Universidad de Cantabria, Edificio Ingeniería de Telecomunicación, Plaza de la Ciencia, Santander, 39005 Cantabria, Spain; dgomez@tlmat.unican.es; 2 Institute for Communication Systems (ICS), University of Surrey, James Clerk Maxwell Building, Guildford, Surrey, GU2 7XH Guildford, UK; t.elsaleh@surrey.ac.uk; 3 Fraunhofer FOKUS, Kaiserin-Augusta-Allee 31, 10589 Berlin, Germany; ronald.steinke@fokus.fraunhofer.de; 4 NEC Europe Ltd., Kurfürsten-Anlage 36, 69115 Heidelberg, Germany; flavio.cirillo@neclab.eu

**Keywords:** Internet-of-Things, testbed, federation, semantic, ontology, proof-of-concept

## Abstract

The Internet-of-Things (IoT) is unanimously identified as one of the main pillars of future smart scenarios. The potential of IoT technologies and deployments has been already demonstrated in a number of different application areas, including transport, energy, safety and healthcare. However, despite the growing number of IoT deployments, the majority of IoT applications tend to be self-contained, thereby forming application silos. A lightweight data centric integration and combination of these silos presents several challenges that still need to be addressed. Indeed, the ability to combine and synthesize data streams and services from diverse IoT platforms and testbeds, holds the promise to increase the potentiality of smart applications in terms of size, scope and targeted business context. In this article, a proof-of-concept implementation that federates two different IoT experimentation facilities by means of semantic-based technologies will be described. The specification and design of the implemented system and information models will be described together with the practical details of the developments carried out and its integration with the existing IoT platforms supporting the aforementioned testbeds. Overall, the system described in this paper demonstrates that it is possible to open new horizons in the development of IoT applications and experiments at a global scale, that transcend the (silo) boundaries of individual deployments, based on the semantic interconnection and interoperability of diverse IoT platforms and testbeds.

## 1. Introduction

The Internet-of-Things (IoT) is recognized as one of the technologies that will enable the metamorphosis of life, business, and the global economy in the future [1]. The IoT vision has been expanded to include pervasive two-way communication and an improved synergy between the digital and physical world. This ability to allow interaction with the surrounding environment is seen as the catalyst for realizing Weiser’s grand vision of ubiquitous computing—ubiquitous computing is positioned as the opposite of virtual reality. Virtual reality puts people inside a computer-generated world, whereas ubiquitous computing forces the computer to live out here in the world with people [2].

However, despite the growing number of IoT deployments (smart houses, smart cities, smart grid, etc.), the majority of IoT applications tend to be self-contained, thereby forming application silos [3]. This mainly results in devices and protocols that are tuned with respect to the enabled applications and businesses to which they are offering their services [4] and cannot be reused. For example, the same temperature sensor used in home automation might not be used in ambient assisted living. It is highly unlikely that a sensor or a node from one vendor-specific application (usually referred to as IoT vertical) is also available to other applications. This is partly due to the fractured research and innovation efforts by various bodies and organizations, causing ossification of applications and hampering the scalability of services offered by vendors and service providers.

The vision of integrating IoT platforms and associated silo applications is bound to several scientific challenges, such as the need to aggregate and ensure the interoperability of data streams stemming from them. The convergence of IoT with cloud computing is a key enabler for this integration and interoperability. It facilitates the aggregation of multiple IoT data streams so that it is possible to develop and deploy scalable, elastic and reliable applications that are delivered on-demand according to a pay-as-you-go model. During the last 4–5 years several efforts towards IoT/Cloud integration have been proposed [5,6] and a wide range of commercial systems (e.g., Xively [7], ThingsWorx [8], ThingsSpeak [9], Sensor-Cloud [10]) are available. These cloud infrastructures provide the means for aggregating data streams and services from multiple IoT platforms. Moreover, other initiatives such as Next Generation Services Interface (NGSI) [11] promoted by Open Mobile Alliance (OMA) or Hyper/Cat [12] funded by InnovateUK [13] focus on the homogenization of interfaces enabling web access to data produced by IoT deployments. However, they are not fully sufficient for alleviating the fragmentation of IoT platforms. This is because they emphasize on the syntactic interoperability (i.e., homogenizing data sources, interfaces and formats) rather than on the semantic interoperability of diverse IoT platforms, services and data streams. They intentionally enforce no rules about metadata naming. Using a reference ontology is one of the currently explored possibilities for having interoperable systems.

Recently, several IoT projects [14] have started to work on the semantic interoperability of diverse IoT platforms, services and data streams. To this end, they leverage IoT semantic models as a means of achieving interoperable modeling and semantics of the various IoT platforms. This is also the pursued approach of the FIESTA-IoT project [15]. The main goal of the FIESTA-IoT project is to open new horizons in the development and deployment of IoT applications and experiments at an EU (and global) scale, based on the interconnection and interoperability of diverse IoT platforms and testbeds.

This paper presents a proof-of-concept (PoC) implementation of the FIESTA-IoT architecture that federates two different IoT experimentation facilities by means of semantic-based technologies. The specification and design of the implemented system and information models will be described together with the practical details of the developments carried out and its integration with the existing IoT platforms supporting the aforementioned testbeds. Main contributions from the paper are the actual implementation of the modules responsible for the annotation, following a common lightweight ontology, of resources and observations, as well as the development and integration of the components that enable testbed-agnostic access to the sensors belonging to the two testbeds that participate in the federation. Specification of the architecture and ontology that underlay the implemented system is a sizeable discussion by itself, so they are only briefly sketched in the paper for the sake of self-contention. Overall, the system described in this paper demonstrates that it is possible to open new horizons in the development of IoT applications and experiments at a global scale, that transcend the (silo) boundaries of individual deployments, based on the semantic interconnection and interoperability of diverse IoT platforms and testbeds. In this sense, the second contribution from the work described in this paper is actually the implementation of two applications leveraging the resulting interoperable federation to access data from both testbeds and to provide value-added services, namely visualization and knowledge extraction, on top of it.

The remainder of the paper is structured as follows: Section 2 presents a review of related work. It will briefly discuss the shortcomings of existing ontologies for its application as basis for the semantic interoperability of the underlying testbeds as well as highlight the limited number of actual semantically-enabled IoT platforms in contrast with the growing number of IoT-related ontologies. The functional specification of the components that have been implemented will be introduced in Section 3, with special emphasis on their interfaces towards the rest of the modules. The practical details of the components’ implementation will be summarized in Section 4. Section 5 describes the two applications developed as a means of validating the benefits of the federated infrastructure. They focus on twofold target: (i) demonstrate access to data and services from multiple IoT testbeds; and (ii) prove support for application portability across testbeds. Finally, conclusions from the work carried out will be derived in Section 6 together with the outline of functional and non-functional extension of the PoC implementation described in the paper.

## 2. Related Work

It is becoming clearer that existing multiple parallel IoT platforms have to converge towards offering seamless, global and linked services to experimenters and users. There is a need for solutions enabling the mechanisms for adapting the already existing IoT infrastructures to provide a common and portable way of sharing their devices and the data generated. One of the motivations of the PoC platform described in this article is to prove the automation of the deployment of services/applications over heterogeneous IoT domains.

Semantics plays a key role aligning the descriptions of the various IoT entities from various testbeds. Being able to reach the abstraction level required for an IoT ontology is challenging. Nowadays, there are a plethora of options which try to represent IoT devices and the ecosystem around them, but still is difficult to find one that fulfills all requirements. Probably, the most widely used ontology is the Semantic Sensor Network (SSN) Ontology [16] that covers sensing, but does not take actuating or other realms of IoT into account. Moreover, this ontology is very complex to use at its full extend and is typically used as a baseline reference. The IoT-Lite ontology [17] uses the SSN as a basis and adds Architectural Reference Model (ARM) [18] key concepts to provide a more holistic IoT model. The adopted solution within FIESTA-IoT has been to reuse these generic ontologies and extend them wherever required to meet the requirements identified for the federation of IoT testbeds.

Other works that are pursuing parallel objectives and that are worth mentioning are OneM2M [19] or IoT-O [20]. OneM2M, as an international partnership project of standardization bodies, is defining a standard for M2M/IoT-communications. The actual release is lacking semantic description of resources that will be addressed as one major point in the next release. The IoT-O ontology aims to unify different ontologies in the IoT landscape and consists of eight different ontologies. It is actively maintained and connected to other standardizations like OneM2M. While many similarities can be found with the FIESTA-IoT ontology, geo-location of resources and observations is not addressed and the virtual entity concept which is central for the IoT-A ARM is not properly covered. Moreover, to the best of our knowledge no major IoT platforms have already adopted it for semantically handling its resources and observations. Other ontologies are focusing on either specific sub domains like the Sensor Web for Autonomous Mission Operations (SWAMO) ontology [21] that concentrates on marine interoperability or not specifically defined for the IoT domain like GoodRelations [22] that is dealing with products but can be taken into account in the industrial IoT area.

Regarding the platforms federation and interoperability support, some research initiatives have been focusing either on middleware frameworks or on the modeling of related knowledge. In the scope of the Fed4FIRE project [23] efforts are made to define a homogeneous way of describing heterogeneous resources within federated testbeds. Starting under the umbrella of Open-Multinet (OMN) [24] and now within the W3C Federated Infrastructures Community Group, they are working towards a set of upper ontologies to describe federated infrastructures and their underlying resources. These ontologies will support a number of use cases to semantically manage the whole life cycle of a resource: discovery, selection, reservation, provisioning, monitoring, control, termination, authentication, authorization, and trustworthiness. They are also providing tools for translating between various ontology. Besides, Fed4FIRE is working on the use of OMF Measurement Library (OML) [25] as a universal mechanism to send measurement data requested by an experiment which could be running on multiple nodes and testbeds. Although the data is still not semantically annotated it offers a way to collect all the measurement data using a generic and flexible software framework. All in all, Fed4FIRE efforts have not focused much on the IoT domain thus overseeing important limitations that makes it difficult to seamlessly apply the solutions they have developed for IoT testbeds.

SOFIA2 [26] is a middleware that extends the results of the EU project SOFIA. It allows the interoperability of multiple systems and devices, offering a semantic platform to make real world information available to smart applications. Open IoT [27] looks at providing a management environment for IoT applications making use of the Sensing-as-a-Service paradigm, along with ontologies for representing the various IoT interconnected-objects. The ontologies defined are based on SSN and SPITFIRE [28].

Project Haystack [29] is an open source initiative to streamline working with data from the IoT. They standardize semantic data models and web services with the goal of making it easier to unlock value from the vast quantity of data being generated by the smart devices that permeate our homes, buildings, factories, and cities.

The IoT Toolkit [30] is an open source project to develop a set of tools for building multi-protocol Internet of Things gateways and service gateways that enable horizontal co-operation between multiple protocols and cloud services.

In this sense, the differential aspect of FIESTA-IoT project is that it is taking a holistic approach, considering the semantic federation of testbeds as a whole, vertically and horizontally integrated, and independently of the environment (smart city, health, vehicles, etc.) they are controlling.

## 3. System Specification

### 3.1. IoT Testbed Federation Abstract Reference Architecture

Before delving any deeper into the actual proof-of-concept system specification, it is important to briefly describe the overall FIESTA-IoT platform concept and the main considerations adopted for achieving the interconnection and interoperability of diverse IoT platforms and testbeds. The PoC system implementation that is described in the following sections integrates the key subset of features that shape the baseline of the complete system. This baseline will be progressively extended by adding further components and functionalities as defined by the reference architecture.

#### 3.1.1. IoT Testbeds Federation Concept

The main aim in the FIESTA-IoT federation is to enable an experimentation-as-a-service (EaaS) paradigm for IoT experiments. However, instead of deploying yet another physical IoT infrastructure, it will enable experimenters to use a single EaaS application program interface (API) for executing experiments over multiple existing IoT testbeds that are federated in a testbed agnostic way. Testbed agnostic implies the ability to expose a single testbed that virtualize the access to the underlying physical IoT testbeds. Experimenters will be therefore able to learn the EaaS API once, and accordingly use it to access data and Resources from any of the underlying testbeds.

To this end, the testbeds willing to participate in the federation will have to implement the common standardized semantics and interfaces that are being defined within the FIESTA-IoT project. This will enable the FIESTA-IoT meta-platform to access their data, resources’ and services’ descriptions and other low-level capabilities.

**Figure 1:**
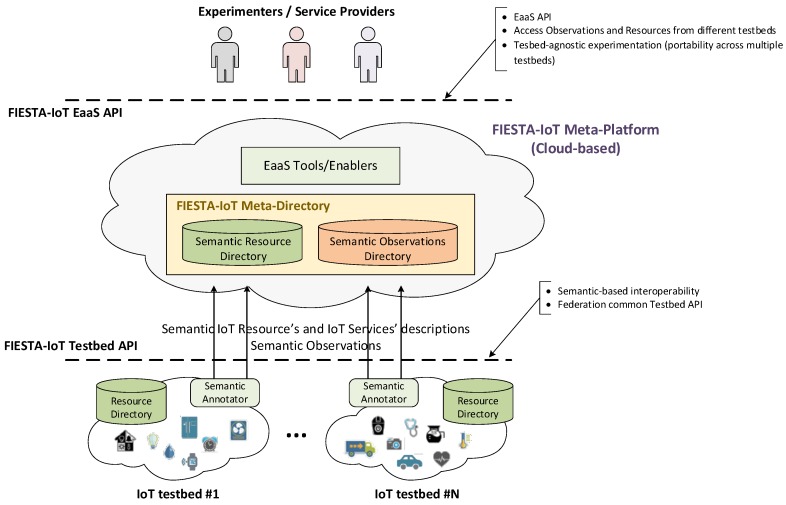
FIESTA-IoT testbed federation concept overview.

As can be seen in Figure 1, the central component of the FIESTA-IoT meta-platform will be a directory service (so-called FIESTA meta-directory), where resources from multiple testbeds will be registered. In the same way, the observations produced by them will be also stored. This directory will enable the dynamic discovery and use of resources (e.g., sensors, services, etc.) from all the interconnected testbeds.

The key concept behind the federation of IoT testbeds is the specification of a common testbed API that will comprise the interfaces to carry out the registration of the testbed resources as well as pushing the observations to the meta-platform. Besides the actual technologies used for implementing these interfaces, the main feature that underlies the FIESTA-IoT Testbed API is the fact that the information is exchanged in a semantically annotated format. In this sense, federated testbeds will have to implement their own semantic annotators, by means of the transformation of the information they handle internally to a common semantic ontology, defined by the FIESTA-IoT meta-platform. Different Resource Description Framework (RDF) representation formats (i.e., RDF/XML, JSON-LD, Turtle, etc.) are supported as long as the common ontology is used.

#### 3.1.2. IoT Testbeds Federation Methodology

A primary decision of the FIESTA-IoT project was to take as reference the IoT ARM as defined in the IoT-A project [18]. This choice has particularly resulted in the observation of the domain model and the information model defined in the ARM. The domain model identifies the key concepts that appears in an IoT environment and the relations between these concepts. The information model defines a meta-model of how to structure information in IoT platforms.

The second main design decision is the use of semantic technologies to support the interoperability between heterogeneous IoT platforms and testbeds. The first step towards a testbed federation is the use of a common language and the definition of relationships between concepts. The taxonomies and ontologies makes it possible to seamlessly deal with data from different sources.

Finally, the third condition to take into consideration is the other enabler for a federation, that is, the means for the interaction between diverse (and potentially heterogeneous) testbeds. FIESTA-IoT aims at providing a blueprint experimental infrastructure, tools, techniques, processes and best practices enabling IoT testbed/platforms operators to interconnect their facilities in an interoperable way.

Thus, the FIESTA-IoT architecture must focus on defining a canonical set of concepts which all IoT platforms can easily adopt. The adoption of these essential concepts should only require from underlying testbeds a straightforward tuning of the models that they handle internally.

The foremost aspect that these choices have implied is that a FIESTA-IoT ontology has been defined to rule the semantic annotation of the core concepts that compose the aforementioned Domain and Information Models. These core concepts are:

- The resource: is a “computational element that gives access to information about or actuation capabilities on a physical entity”.
- The virtual entity: is a “computational or data element representing a physical entity”.
- The IoT Service: is a “software component enabling interaction with resources through a well-defined interface. It can be orchestrated together with non-IoT services (e.g., enterprise services). Interaction with the service is done via the network”.

These concepts conform the baseline for representing the devices and overall IoT infrastructure. However, there is still a major concept that is not tackled within the ARM models. This concept relates to the actual data that is gathered by the devices and offered through the services that expose them. Namely, it is the observation concept:

- An observation is a piece of information obtained after a sensing method has been used to estimate or calculate a value of a property of an Entity.

Linked to this concept and its relation to the entity one through the property idea, another important aspect that has been also addressed during the construction of the FIESTA-IoT ontology is the definition of a taxonomy that sets a common ground for the description of the physical phenomena and units of measurement captured in the observations.

**Figure 2:**
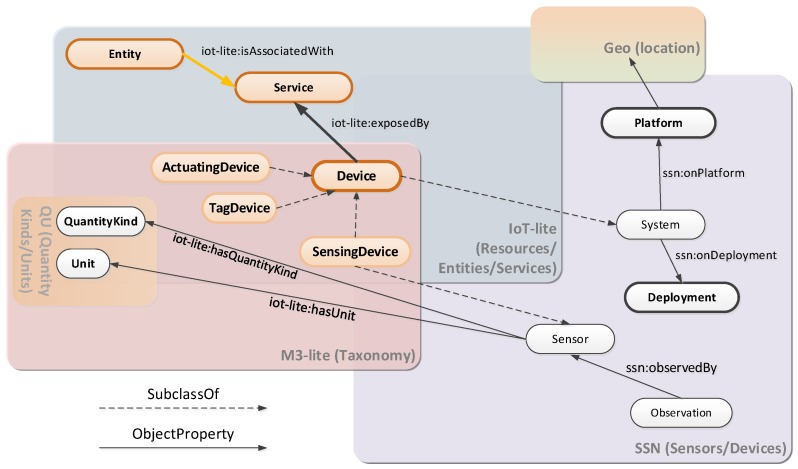
FIESTA-IoT ontology key concepts overview.

Figure 2 shows a high-level overview of the main concepts which are managed in the ontology defined. It is important to note that just the key concepts are included in Figure 2 but several other ontology classes and properties have been defined. However, the complete description of the FIESTA-IoT ontology is out of the scope of this paper. Hence, only the relevant aspects necessary to understand the approach followed to implement a semantically interoperable federation of IoT experimentation facilities are highlighted.

It is important to emphasize that this ontology is the baseline for the interoperability of the heterogeneous testbeds and IoT platforms that are expected to be federated in the FIESTA-IoT meta-platform. The different testbeds have to converge for participating in the federation and they use this ontology as the reference for this convergence. Precisely this is the main reason why the ontology has been kept simple as a design decision.

Another important design consideration has been the re-use, as much as possible, of already well-established ontology concepts. In this sense, for the core ARM concepts, the FIESTA-IoT ontology has taken the IoT-lite ontology [17], a lighter version of the IoT-A ontology [31] developed as a part of EU FP7 FIWARE project [32]. However, many updates have been performed to IoT-lite so that it can be reused in FIESTA-IoT project. For the observations aspect which was not correctly captured by IoT-Lite, the SSN ontology [33] has been used. This ontology is used especially to describe Resources and Observations, and related concepts. Finally, the phenomena and units of measurement related concepts have been incorporated to the FIESTA-IoT ontology through the M3-Lite taxonomy [34]. This taxonomy has been created by integrating and aligning already existing ontologies in order to homogenize the existing scattered environment in which a quite large number of similar ontologies define the same concepts in an overlapping manner.

### 3.2. Proof-of-Concept System Functional Specification

As has been previously mentioned, the PoC platform integrates the key subset of features that are necessary to validate the federation paradigm and that shape the baseline system on top of which additional components (offering further functionalities) will be added. As has been shown in Figure 1, the envisaged FIESTA-IoT meta-platform will include two directories. The first one allowing testbed agnostic retrieval of sensors’ observations, and the second one enabling federated discovery and access to underlying testbeds’ resources and services.

While the final implementation of the meta-platform will include the two repositories, the developments that have been carried out and integrated for the PoC system that is described in this article are restricted to the resource directory. In this sense, since it is not part of the contributions we are presenting in this article, the semantic observation directory is not further described.

**Figure 3:**
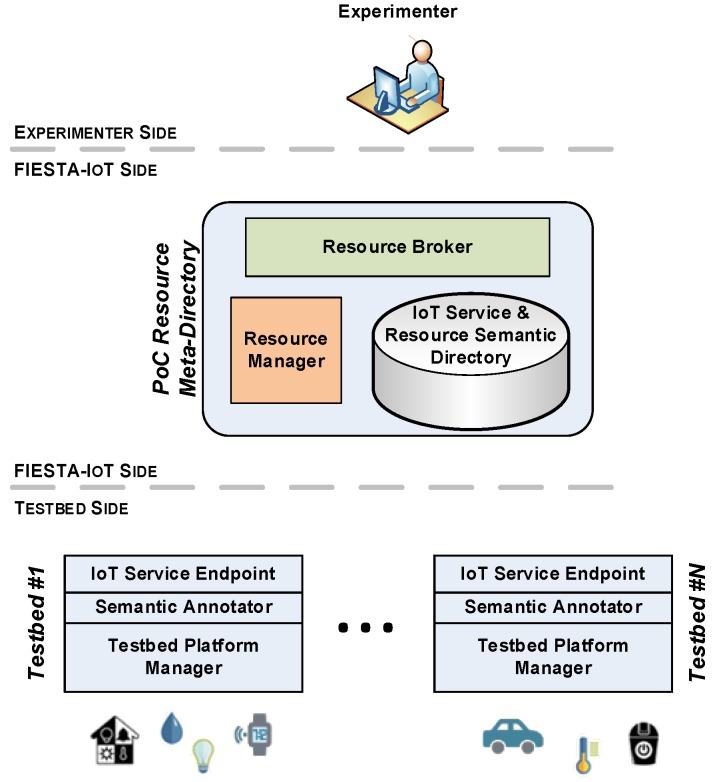
PoC system functional architecture.

Figure 3 shows the functional architecture of the actually implemented PoC system. As it has focused on the Resource-based federation realm, out of the complete FIESTA-IoT platform, only the following functional components have been considered for the scope of the PoC system:

- Resource manager (RM): this component is the responsible for validating the semantic resource descriptions that the federated testbeds use for the registration of their resources and for implementing the final adaptations to these resource descriptions before storing them.
- Resource broker (RB): this component implements the interfaces offered by the IoT-service/resource registry to the experimenter.
- IoT service & resource semantic directory (SRD): this component is the triple-store that keeps the IoT service and resource descriptions.

While most of the functionalities needed to establish the testbeds federation are supported by the meta-platform components, testbeds willing to be part of such federated system have to fulfill a minimum set of requirements. These requirements can be basically summarized as a necessity to comply with the FIESTA-IoT semantic model. Thus, it is necessary for the testbeds to integrate within their architectures a component that deals with the transformation between the models they handle internally and the common FIESTA-IoT model. This component, as can be seen in Figure 3, is the semantic annotator. It has been integrated just below the testbed IoT service endpoints. These endpoints are basically the interfaces that allow access to the APIs exposed by the underlying resources. The semantic annotator is responsible of taking the information (whether it is a resource description or an observation), expressed according to the testbed proprietary model, as it is served by the native testbed platform (condensed in Figure 3 as testbed platform manager) and transforming it so that it complies with the FIESTA-IoT semantic model.

**Figure 4:**
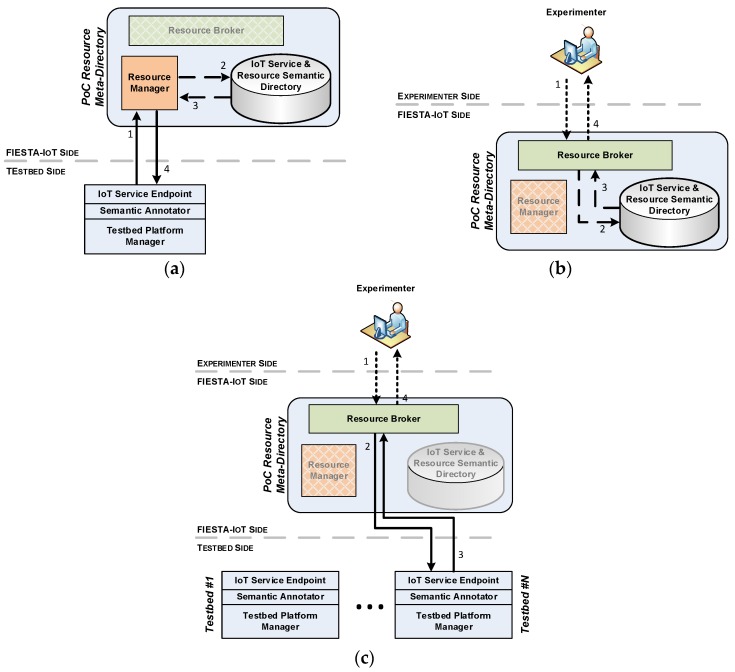
PoC system functional use-cases. (**a**) Testbed Resource registration; (**b**) Experimenter Resource look-up; (**c**) Experimenter IoT Service invocation.

Figure 4 shows the main functional use-cases that are supported by the PoC system. Experiments making use of the implemented PoC system will basically be able to first query it for discovering the devices fitting the experiment’s needs (cf. Figure 4b).

These queries can look up for devices based on their location and/or the phenomena that they are able to monitor. The responses to these queries will not depend on the testbed to which the matching resources belong but on the criteria embedded within the query. The RB will forward these queries to the SRD which in turn produces the corresponding responses that will contain the bindings as per the resources registered therein. However, the main service demanded from an IoT testbed is not to provide information about which sensing capacities it has, but to actually serve the data that these IoT devices are producing. Hence, to fulfil the aforementioned baseline functionalities, the PoC system also establishes the mechanisms facilitating the access to the observations gathered by the underlying testbeds’ IoT devices. In this sense, a key aspect of the semantic model used for the description of the testbeds’ resources is its ability to link the different IoT domain model concepts into a connected graph. The FIESTA-IoT platform in general and the PoC system in particular takes benefit of this and makes it possible that at the same time that Resources are discovered, the IoT services that expose them are also revealed. Thus, experiments will be able to extract from the responses to their resources look-up queries, the IoT services that they have to invoke to access the observations captured by the corresponding IoT devices (cf. Figure 4c). In this case, the RB will intercept the query made and redirect it to the relevant testbed endpoint. It is important to highlight that both the discovery of the resources matching the specified criteria, and the invocation of the associated IoT services are centralized in the PoC platform. The necessary adaptations to support multiple underlying testbeds are internally handled, and are transparent to the experimenter who has the impression of using just one single meta-platform.

Still, results from the discovery process and subsequent access to exposed IoT services, will depend on the resources that testbeds had registered beforehand. In this sense, the federation begins before it is actually used in an experiment. Federated testbeds will effectively initiate it as soon as they start populating the SRD with the annotated versions of their resources’ descriptions (cf. Figure 4a). RM takes the semantically annotated resource descriptions and, after checking its correctness (only syntactic validation is currently performed), it registers it in the SRD. Upon successful storage on the Directory, the result of the registration is notified back to the testbed. Next we will further describe each of the components that are part of the PoC meta-platform specification.

#### 3.2.1. Resource Manager

This component exposes the single entry point for all the testbeds to register their resources’ descriptions. Its main role is to homogenize the descriptions received from the different testbeds. After syntactically checking the annotated descriptions and guaranteeing that they are compliant with the FIESTA-IoT ontology, the RM “impersonates” all the resource descriptions. This process basically consists of overwriting the bindings that point to the original testbeds’ domains included in the annotated resource descriptions. These bindings are transformed to the common meta-platform domain so that every entity identifier and/or IoT service endpoint, independently of which testbed they belong to, are exposed as if they belonged to a unique graph, namely the federation graph.

Once the necessary adaptations to the resource descriptions have been done and internally recorded for future use by the RB, the RM stores them into the SRD. Therefore all the semantically annotated descriptions generated by the testbeds are stored in the SRD following the testbed agnostic paradigm followed within FIESTA-IoT.

While the communication interface between the RM and the SRD will be based on semantic requests, the interface with the testbeds is based on standard HTTP encapsulating semantically annotated documents.

#### 3.2.2. Resource Broker

This component exports the FIESTA-IoT platform functionalities to the experimenter or end-user. It supports both the resource look-up and the IoT service invocation use cases.

In the first case it basically compiles the responses acquired from the SRD, according to the experiment constraints with respect to data format, flow rate, etc. However, its main role refers to the latter case. Resource descriptions discovered through the resource look-up have been impersonated during the registration process by the RM so that they are all bound to the meta-platform domain. Thus, it is necessary to make the inverse transformation in order to redirect each IoT Service invocation to the appropriate resource at the corresponding testbed. The RB leverages the record of transformations set by the RM for this. In this sense, the RB will basically proxy the experimenter IoT service queries.

#### 3.2.3. IoT Service & Resource Semantic Directory

The SRD acts as the repository for the resource descriptions registered by the underlying testbeds. It offers the interfaces for the management of the descriptions, i.e., lookup, update or removal. While the RM and RB exposes non-semantic interfaces, the SRD is able to resolve semantic queries over the stored triples. The database where the semantic descriptions are stored can be either full triple store or SQL database offering a semantic endpoint.

As the testbeds are populating the SRD with their information, the SRD might be replicating all the information available in the testbeds. This behavior has sense when the testbed is not semantic-ready, that is, it does not implement the components to support the storage of semantic data (i.e., triple store database) or the query engine to access it. However, the design made also considers testbeds that are able to natively support semantic representation of their resources. This kind of testbeds will have their own SRD. Resource look-up queries are managed in a distributed way so that requests are not only answered considering the information stored on the FIESTA-IoT SRD, but they would be also redirected to the SRDs of the testbeds with this directory. Supporting this distributed discovery functionalities to answer a look-up request implies the participation of the RB and the RM. They will be in charge of interacting with the central SRD and the remote testbeds’ repositories and generating (if there is any) the response to the query.

#### 3.2.4. IoT Resource and Observations Semantic Annotators

Semantic annotators are responsible for translating data and metadata expressed in a non-semantic format (e.g., JSON) into a semantic RDF format. Any data or resource description provided by a testbed to the meta-platform must be expressed in a semantic manner and must be compliant with the FIESTA-IoT ontology.

The first step to be carried out when a testbed wants to be part of the federation is the registration of its devices. Therefore, one of the main requirements is the adaptation of the descriptions provided to a format and language which is understood by the FIESTA-IoT system.

**Figure 5:**
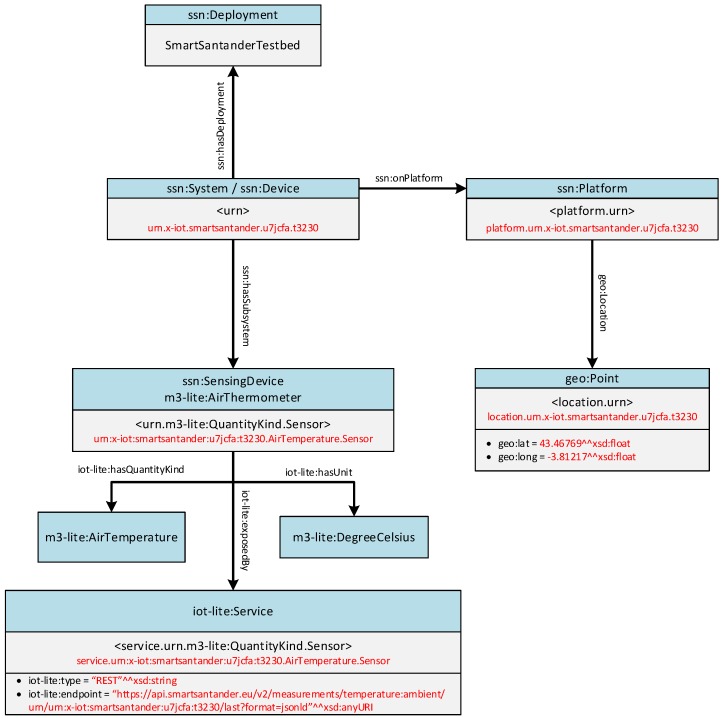
Semantic graph of an IoT resource description.

Out of the complete FIESTA-IoT ontology, the minimum information that a semantically described resource has to include is shown in Figure 5. The graph shows the semantic entities with their associated classes (blue boxes), their data properties in case they have them (bullet points) and the relationship between the various entities. For the sake of exemplification, the graph has been particularized for a SmartSantander resource, whose entities’ identifiers and literals are noted in red.

In this sense, the semantic annotator has to retrieve the necessary information in the internal proprietary format that is used within the testbed (e.g., urn, location, sensed phenomena, etc.) and create with it an RDF document using the concepts and relationships defined by the FIESTA-IoT ontology as it is exemplified in Figure 5.

**Figure 6:**
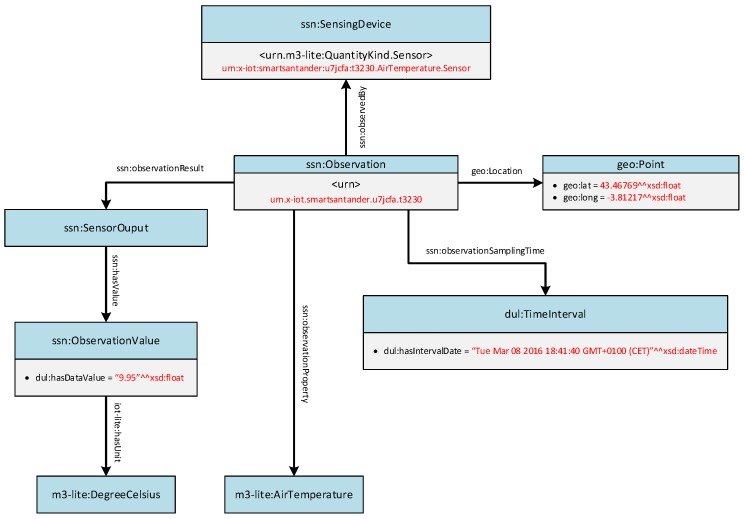
Semantic graph of an observation.

Semantic annotator does not only have to create the semantic resource descriptions but also has to transform the measurements that these resources gather. Similarly to what was necessary for the resource description, testbeds have to provide a minimum set of semantically annotated information to be compliant with FIESTA-IoT requirements. Following the same format as Figure 5, the graph shown in Figure 6 depicts an arbitrary observation.

From the above, each testbed should make an internal analysis of their own description for resources and observations, and identify which attributes are the corresponding match with the defined FIESTA-IoT entities. Once this task has been fulfilled they have to generate an annotated document, which will feed the FIESTA-IoT meta-platform. In order to make it easier for testbeds to become part of FIESTA-IoT federation, FIESTA-IoT supports the most common semantic description formats, like JSON-LD, Notation3 (N3), RDF/XML, OWL/XML, or TURTLE. Besides, all the annotators developed by the FIESTA-IoT first-parties will be completely available for external users so that they can tailor their corresponding formats to that of FIESTA-IoT, without having to start the development of its own semantic annotator from scratch.

## 4. PoC Implementation

As has been stated in the Introduction, the main contribution described in this article is the implementation of the system that the authors have carried out. Following the specification of the modules previously depicted, the implementation and integration of them into a PoC system has been accomplished in order to demonstrate the viability of the meta-platform federation vision.

The implementation and validation of the integrated system has been done over two existing testbeds. The first of them is the SmartSantander testbed. SmartSantander is an experimental test facility for the research and experimentation of architectures, key enabling technologies, services and applications for the IoT in the context of a city (the city of Santander located in the north of Spain). The infrastructure deployed includes a capillary network covering a wide area of the city made up of 12,000 diverse IoT devices. This set of devices produces up to 300,000 Observations per day, which are central to the provision of added-value services to the citizens of the city of Santander while attracting researchers to design, create and run their experiments. The second one is the SmartICS testbed at University of Surrey. SmartICS is an IoT platform for enabling smart offices. It consists of devices called “IoT nodes” which are fixed on every office desk in the building. Each IoT node is made up of a Plogg device combined with an in-house sensor suite module. Its purpose is to capture a range of information relating to each desk, which include power consumption, ambient temperature, light intensity, noise and presence. Further details about both of them can be found in [35,36,37,38]. In the following sections, the insights of the development of each of the system components will be presented.

### 4.1. Semantic Resource Meta-Directory Implementation Details

From the architecture and design pinpoint the resource meta-directory has been split into three different functional components. On the other hand, for the actual implementation all the functionalities have been integrated into a single module around the SRD, which addresses all the resource meta-directory functionalities.

The SRD exposes a RESTful API for registering, managing and querying resources. The operations below will partly or wholly have the uniform resource locator (URL) structure below:

http://{server_host}/srd/{endpoint_name}/{repository_id}/{resource_id}

where:

- server_host: is the IP address or hostname of the server. This will also include the port number if other than the default HTTP port 80 is used.
- endpoint_name: name of the endpoint that is used. This can be either registry or sparql.
- repository_id: is the ID of the target repository on the server. The server might have one or more repositories.
- resource_id: is the id of the resource or entity.

There are two endpoints that can be targeted in the SRD. The first being the registry, which is used for registration and management (i.e., update and removal) and the other, sparql, exposing a SPARQL endpoint that can be used for the discovery of existing resources. These separated endpoints basically address the different functionalities demanded by experimenters and testbed providers. In this sense, the sparql endpoint represents the northbound interface and is typically used by experimenters. The registry endpoint exposes the southbound interface of the meta-directory and is used by testbed providers.

When it comes to management of resource descriptions, the API makes use of the main HTTP methods (i.e., GET, POST, PUT and DELETE). Specifically, the sparql endpoint allows both GET and POST. In the first case, the look up query is directly included as a parameter in the uniform resource identifier (URI) while in the latter case the SPARQL query is directly embedded into the body of the HTTP request. Analogously, the registry endpoint allows POST, PUT and DELETE methods for respectively registering, updating and removing resource descriptions. When new resources are to be registered or existing ones updated, their semantic descriptions are embedded into the corresponding HTTP request. In this sense, it is important to highlight that the implemented SRD also enables the registration of multiple resources at once.

For RDF descriptions, the SRD is able to manage several formats. Experimenters and testbed providers can specify the format of the descriptions they are sending or receiving by using some header fields in the HTTP request. These header fields are Content-Type for specifying the format of the description embedded into the HTTP request body (i.e., in case of registration or update of new resources), and Accept for specifying the format of the descriptions to be included within the body of the HTTP responses (i.e., when making a discovery). To specify a format, the corresponding internet media type must be used. The formats supported are JSON-LD, Notation3 (N3), RDF/XML, OWL/XML, or TURTLE. In the case of non-RDF responses from SPARQL requests (e.g., SELECT), the formats accepted are JSON, XML, CSV, TSV, or Text.

Concerning the repository_id element of the URL, the implementation allows for the SRD to host multiple repositories. This enables the creation of some kind of hierarchy within the resources. The federation manager can establish, together with the authorized testbed providers, the policies defining how many repositories can be used and how they are organized. Having one excessively large repository might not properly scale when experimenters want to look for meta-platform resources. Although this feature has not been actually used for the PoC federation described in this paper, the architecture that has been defined specifies that testbeds can themselves keep their resources in its own SRD (not using the central instance). These distributed SRDs are subsequently federated through the resource manager module. Additionally, the resource manager can itself create a hierarchy based on domain of interest or location in order to group resources which are closely related, and would generally be queried together. This would optimize the discovery process and improve the system’s scalability. Precisely, concerning the resource discovery and its scalability, the resource broker, being the responsible of this functionality, has been specified and implemented as a completely stateless component. This means that multiple instances of the resource broker can work in parallel serving the demands from the experimenters and solving any bottleneck concerns. Whether there is a single SRD or many of them, every instance of the resource broker module, in collaboration with the resource manager, will be able to solve resource discovery queries coming from the experimenters.

Finally, the SRD implementation has been developed to exploit the linked data paradigm. In this sense, the URIs assigned for identification of “semantic” instances (or individuals) of resources are dereferenceable URIs. This means that they act as valid URLs exposing a web resource, which in this case is the resource. For example, if we have a resource with an ID *IoT-Node-001* that is to be registered at a semantic registry at http://platform.fiesta-iot.eu/srd/registry/ with a repository name *myrepo*, then its URI will be a combination of host name of the SRD, the path, and the resource ID at the end. This becomes the following URL: http://platform.fiesta-iot.eu/srd/registry/myrepo/IoT-Node-001 which can be accessed through an HTTP GET request obtaining as response the resource description of *IoT-Node-001*.

### 4.2. IoT Resource and Observations Annotators Implementation Details

As has been already introduced, semantic annotators run at testbed level. In this sense, their implementation heavily depends on the testbed specific characteristics. In the majority of the cases, testbeds will have their own model and the annotator will have to perform an adaptation. However, it could be the case that a testbed does not handle a resource description per se and then the annotator will have to directly create the semantic description. Depending on the testbed, not only the number of attributes included in the descriptions, but also the naming and format of these attributes vary. FIESTA-IoT ontology defines the set of relevant information that every resource description and observation should contain. Then, it is up to each semantic annotator to map its internally managed attributes to the FIESTA-IoT semantic entities.

Precisely, the two testbeds that have been federated for this PoC case exhibit this heterogeneity. While the SmartSantander testbed has a proprietary model for describing its resources, the SmartICS testbed does not have a parallel document that contains resources descriptions. Hence, the annotator implemented for the SmartSantander testbed played a translator role, while the SmartICS one basically created from scratch the semantic descriptions for its resources.

In order to avoid repetition, this section will only describe the process of semantically annotating the SmartSantander resources. In this sense, the first step in this process relates to the identification of relevant pieces of information within the SmartSantander resource model. The second one is the actual creation of the annotated description which is, indeed, analogous to the complete operation carried out by the SmartICS annotator.

SmartSantander follows a proprietary format based on the utilization of JSON schemas both for resources and observations. The JSON schema for describing the resources is structured in six sections or global properties:

- Identification: set of properties that hosts the minimum identification details for that resource, including the uniform resource name (URN).
- Location: property modelled following the GeoJSON schema [39] that references the position of the sensor node.
- Description: property gathering descriptive human-readable information.
- Service: property containing an array of the sensing capabilities and functionalities of the resource. These capabilities define the information the resource is able to produce considering the physical phenomena it can measure.
- Experimentation: set of properties containing parameters to be used in the low-level experimentation.
- Management: property including status information related to the sensor life cycle (events, etc.)

Out of them, for the PoC, the management and experimentation information are not considered as they do not include any relevant information necessary to fill the properties and instances defined within the FIESTA-IoT ontology.

**Figure 7:**
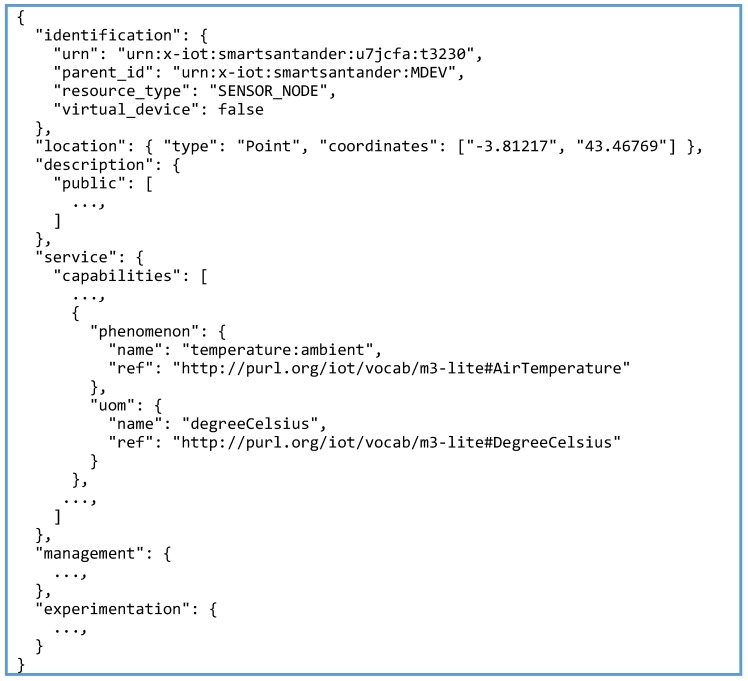
SmartSantander Resource JSON description.

Figure 7 shows an excerpt (some parts of the device description shown in Figure 7 have been intentionally snipped for the sake of concreteness but the complete JSON document can be found in the Supplementary Material accompanying this paper) of an exemplifying description of one of the devices from SmartSantander. One interesting aspect from this description is the reference to the sensing capabilities of the device (only ambient temperature has been kept in Figure 7). In this sense, the approach that has been taken in the SmartSantander annotator for implementing the mapping to the FIESTA-IoT semantic model is to consider that a SmartSantander device is composed by as many sensors as sensing capabilities the device has.

Taking this into account SmartSantander resource descriptions are organized around the ssn:Device class in the FIESTA-IoT ontology. From there, each of the sensing capabilities are considered as an ssn:SensingDevice, with its corresponding quantity kind and unit of measurement. For a better characterization of the sensing capabilities and, by extension, particularizing the ssn:SensingDevice type, we have also mapped the sensing capabilities with the ssn:SensingDevice sub-classes defined in M3-lite. The rest of the entities and properties, such as the associated deployment (ssn:Deployment), the device location (geo:Location), etc. can be directly obtained from the values of the rest of the SmartSantander description properties.

The graph representation of the SmartSantander resource shown in Figure 5, eventually refers to the same device described in Figure 7. The root of the graph (i.e., ssn:Deployment) refers to the testbed to which the device belongs. The URN of the device is used to designate the instance of the ssn:Device class itself. The resource is bound to a physical location through the physical entity to which the device is attached (i.e., ssn:Platform). As aforementioned, one single device might be equipped with several ssn:SensingDevices (as a matter of fact, as can be checked in the Supplementary Material, for Figure 5 to faithfully represent the actual IoT device, not just the excerpted view shown in Figure 7, it should not only include an m3-lite:AirThermometer that measures the ambient temperature, but also an m3-lite:ElectricalSensor that measures the battery level remaining, and an m3-lite:LightSensor that gathers the information about the illuminance perceived in the ambient). Note that the naming of these elements matches the ones defined in the M3-lite taxonomy. As could be easily inferred, each of these ssn:SensingDevices is linked to a pair m3-lite:QuantityKind/m3-lite:Unit, which makes a unique bound between the resource type and the physical phenomenon they are collecting information from. Finally, the IoT service that exposes each of the resources (e.g., to retrieve the last value measured) is also annotated. These services are linked to the proprietary REST interfaces defined within SmartSantander to retrieve the information of various physical phenomena a resource can observed.

Regarding the actual software implementation of the annotator, the approach taken was to extend currently available functionalities of the SmartSantander testbed platform manager, developed in Node.js [40]. The new set of modules added serializes the resources’ description following the FIESTA-IoT ontology. These modules are based on RDF-Ext [41], an open source JavaScript library for working with RDF and linked data.

**Figure 8:**
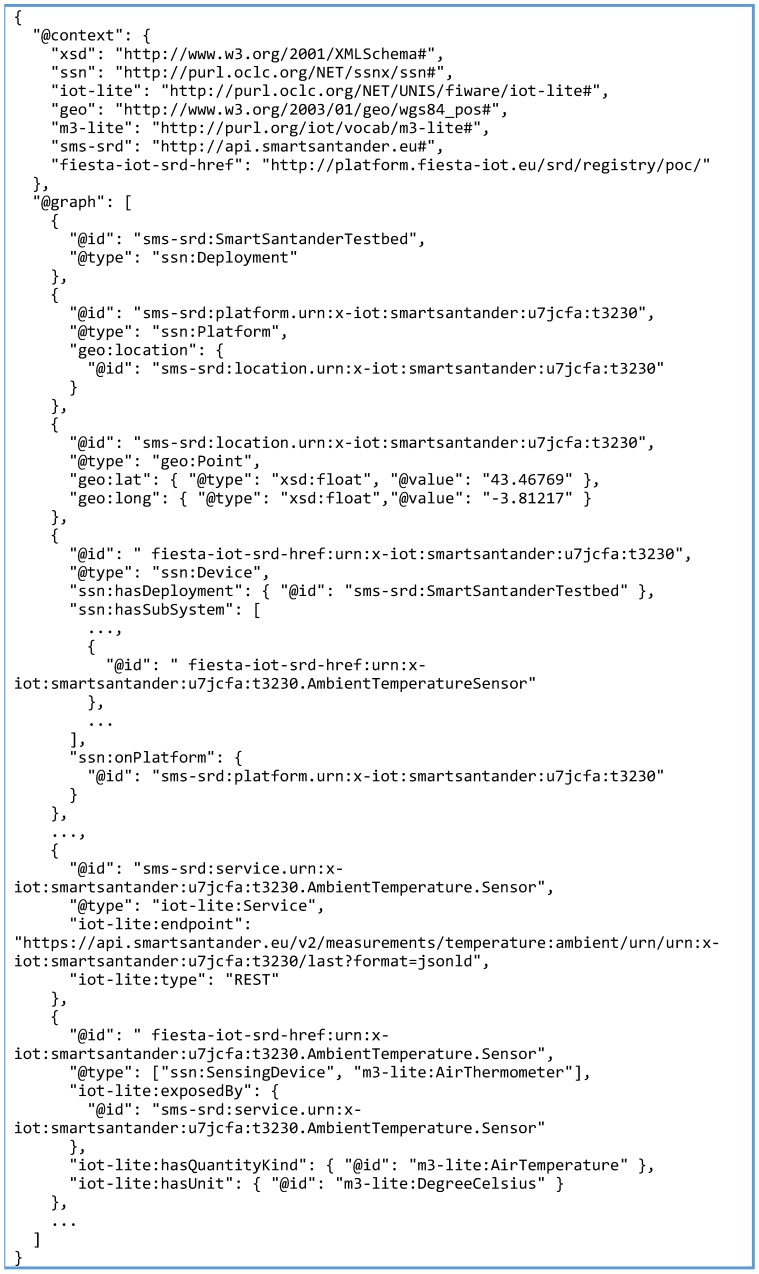
Annotated SmartSantander Resource in JSON-LD format.

This library contains the core classes to handle the RDF model data. The annotator developed within SmartSantander is able to serialize the original resource description in JSON-LD, RDF/XML or Turtle. The annotated version of the SmartSantander resource included in Figure 7 is shown in Figure 8 (some parts of the annotated description shown in Figure 8 have been intentionally skipped for the sake of concreteness but the complete JSON-LD document can be found in the supplementary material accompanying this paper). The device is represented using JSON-LD graph, following the FIESTA-IoT ontology.

**Figure 9:**
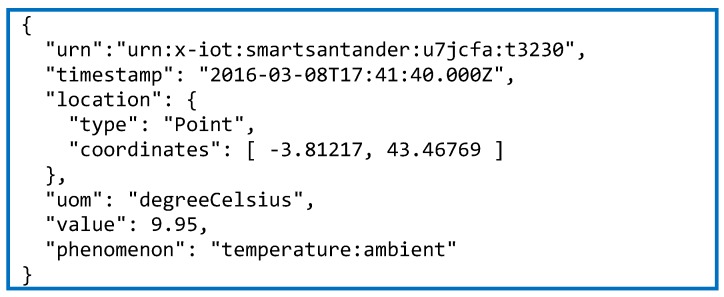
SmartSantander observation JSON description.

**Figure 10:**
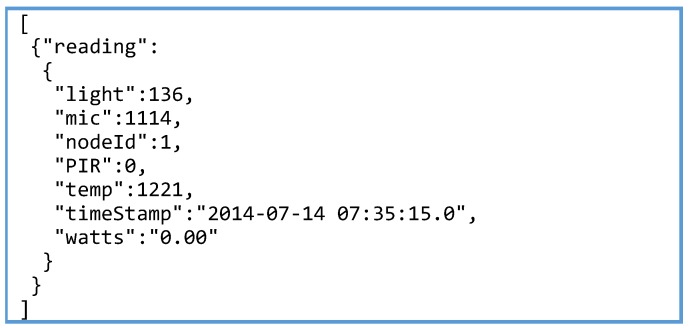
SmartICS observation JSON description.

Once the process for generating the annotated resource descriptions has been presented, next the procedure followed to expose an annotated IoT service endpoint will be described. In this case the process is analogous for the two federated testbeds but small implementation differences applies. Natively, both SmartSantander and SmartICS testbeds returns the information gathered by their devices as Observations that are described in their own proprietary JSON format. Figure 9 and Figure 10 show two examples of SmartSantander and SmartICS observation object format, respectively. As can be seen, the way in which information about the value, phenomenon, unit, timestamp and/or a location is represented is completely different. Therefore, any experimenter willing to get information from both testbeds would need to get familiar with the two models in order to parse and interpret the received observations.

Following a similar approach to the one followed for the annotation of the SmartSantander resources’ description, firstly, the correspondence between the proprietary properties and the FIESTA-IoT ontology were identified. The observation object is considered as an instance of a ssn:Observation class, and gathers all the parameters that shape it. The graph representation of the sample annotated observation shown in Figure 6, eventually refers to the one presented in Figure 9.

**Figure 11:**
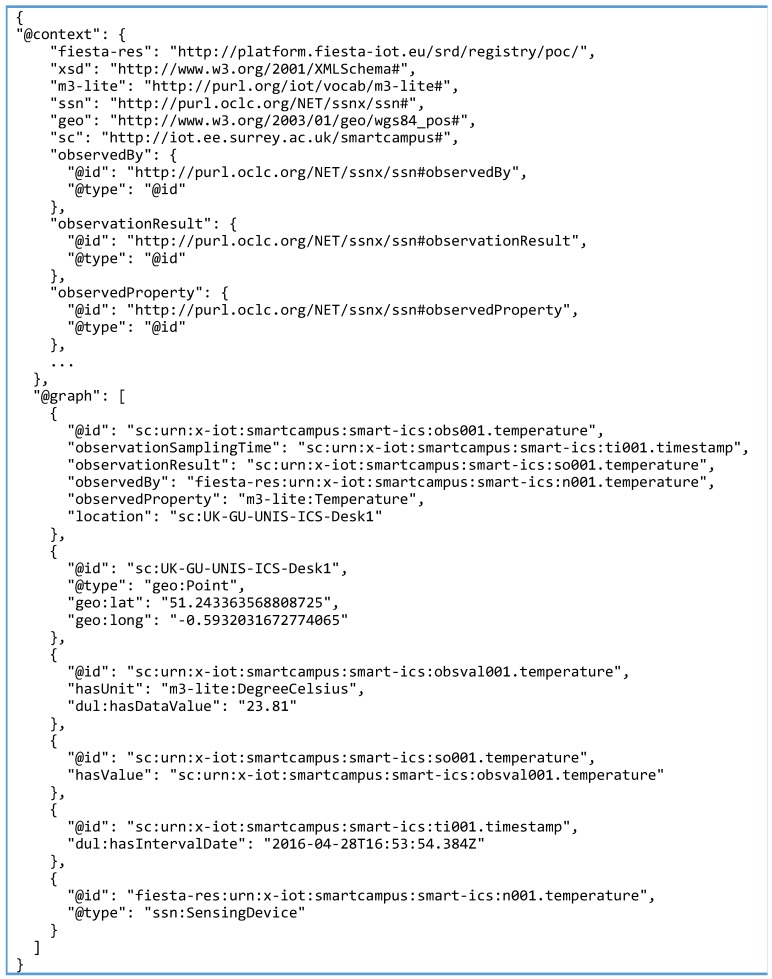
Annotated observation in JSON-LD format.

Also with regards to the resource semantic description, assigning a unique identifier to the annotated observation or any entity linked to it is optional. In any case, if assigned, the URI used for that entity would be not dereferenceable since it is considered that it is uniquely identified by the timestamp and the node generating it (which actually has a dereferenceable URI). Figure 11 shows an annotated observation from one of the SmartICS devices. As can be seen, SmartICS annotator actually provides a specific URI to its observations and related properties’ instances (a semantically annotated observation from SmartSantander annotator is provided as supplementary material accompanying this article. In this case, anonymous instances are used). Still, the device generating the observation (i.e., linked through the ssn:observedBy object property) has the derefenceable URI provided when it was registered at the resource meta-directory.

Regarding the actual software implementation another difference between the two annotators is that the SmartSantander observation annotator also was integrated in such a way that it extends currently available functionalities of the SmartSantander testbed platform manager. On the contrary, the SmartICS annotator is deployed as a proxy between the client and the SmartICS Testbed platform manager. Upon request, the annotator fetches the original data description from the SmartICS data broker, extracts the data and re-annotates it according to the FIESTA-IoT ontology.

Indeed, the design was made in this way to be flexible enough for the testbeds to be able to embed their respective annotators in the most convenient way for them. The meta-platform just demanded semantic interoperability letting some degree of freedom to the approach chosen for carrying out the adaptation.

As described above, the annotation process enables the exportation the data in a standard semantic way. Computationally speaking, we do not believe that this process will introduce any significant overhead, since it will mainly consist in renaming and serializing the entities, values, etc., thus tailoring the testbeds’ resources descriptions and observations according to the FIESTA-IoT ontology. Then, as a result, the most likely overhead is the larger amount of data exchanged through the network. However, this will depend on the information provided by each of the testbeds.

All in all, the main overhead foreseen of the proposed semantic-based platform resides in the management of triple store databases and the execution of inference and reasoning engines. It is worth highlighting that these ones will be run independently from the testbeds. As the amount of data collected increases, the look-up operations might take longer. Nevertheless, by deploying triple store databases in a distributed manner and by parallelizing the access to them, we really envisage to minimize the performance degradation of the overall system.

## 5. Experimenting over Semantically-Federated IoT Testbeds

The testbed Federation created through the implemented PoC system described in previous sections has enabled the validation of the main benefits from the establishment of such virtualized meta-platform. This is the capability for designing and conducting experiments which share, link and produce easily accessible IoT datasets stemming from different IoT testbeds.

The following sections will depict the applications that the authors have developed aiming at demonstrating the unified and easy access to data from different testbeds. Two applications have been developed. The first one focuses on creating a graphical user interface (GUI) for browsing the meta-testbed resources. The second one aims at leveraging data analytics algorithms over the available information to build a set of indicators showing the status of a city. Both applications share a common graphical interface based on a map where the available testbeds are shown. Upon experiment and testbeds selection, the process of gathering and data analysis is triggered. On completion, the results in bulk or previously processed are presented in the map.

### 5.1. Federation Resource Browser Application

The Federation Resource Browser application enables a generic interface for unified data retrieval from the available testbeds. The application offers a graphical interface where the experimenters can select the resources from which they want to retrieve data. Starting from the representation of all the resources in a map, users can set several selection criteria like map boundaries or categories (temperature, traffic, etc.). Once selected the desired resources, data is requested and shown accordingly in the map.

The application consists of a backend server and a GUI. The backend server has been implemented in a Node.js environment and provides a JavaScript-based web-application frontend. Web sockets are used for the communication between the backend server and each client frontend as the way to provide seamless feedback. The backend provides a website that is working as an application. Amongst the programming libraries used, it is worth emphasizing that the links with each of the testbeds and their resources’ service endpoints are established on-demand, following the traditional JavaScript asynchronous manner. This way of proceeding provides a better user experience and a more appropriate management of the remote resources interfaces.

**Figure 12:**
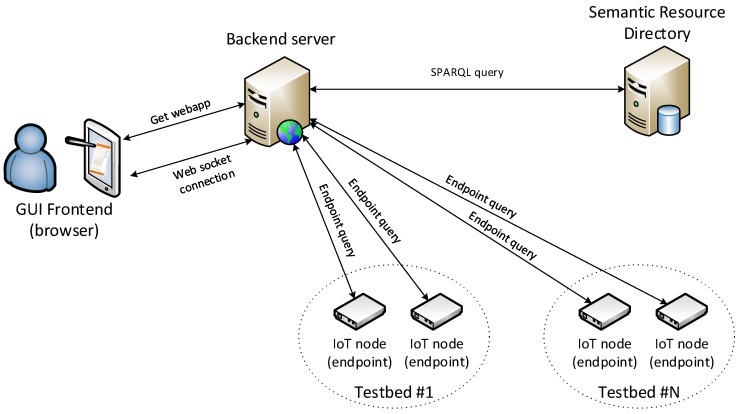
Federation Resource Browser system architecture.

Figure 12 shows the components of the application and the connections between them. First of all, the backend server translates the requests for resource’s data received from the client on the web service interface into SPARQL. Then, it forwards them to the SRD who, in turn, returns the endpoints associated with the resources. The communication between the different entities is based on HTTP. In order to avoid potential cross-site scripting problems, we have used web sockets and delegated the request to the backend. Additionally, the web socket connection is being used to provide a more fluent way of exchanging information within a single request and response. For example, preliminary results are transmitted to show the positions of the resources when they are available. The query and processing times can be also shown in the frontend as soon as they are available. Finally, the progress of querying the endpoints can be visualized to provide useful feedback to the user as the selection can be very broad and the gathering time can be high. As it has been previously highlighted, this operation mode enhances the user experience.

Probably, one of the most demanded use-cases is the request for data from resources filtered based on specific phenomena. The solution implemented is making it possible to retrieve that data following the testbed agnostic paradigm followed in FIESTA-IoT. As every testbed has registered its resources according to FIESTA-IoT ontology, the resources can be accessed through the same category name. Then, the associated sensors from the different testbeds provide the same information model independently of their internal data format, as described in the previous sections.

**Figure 13:**
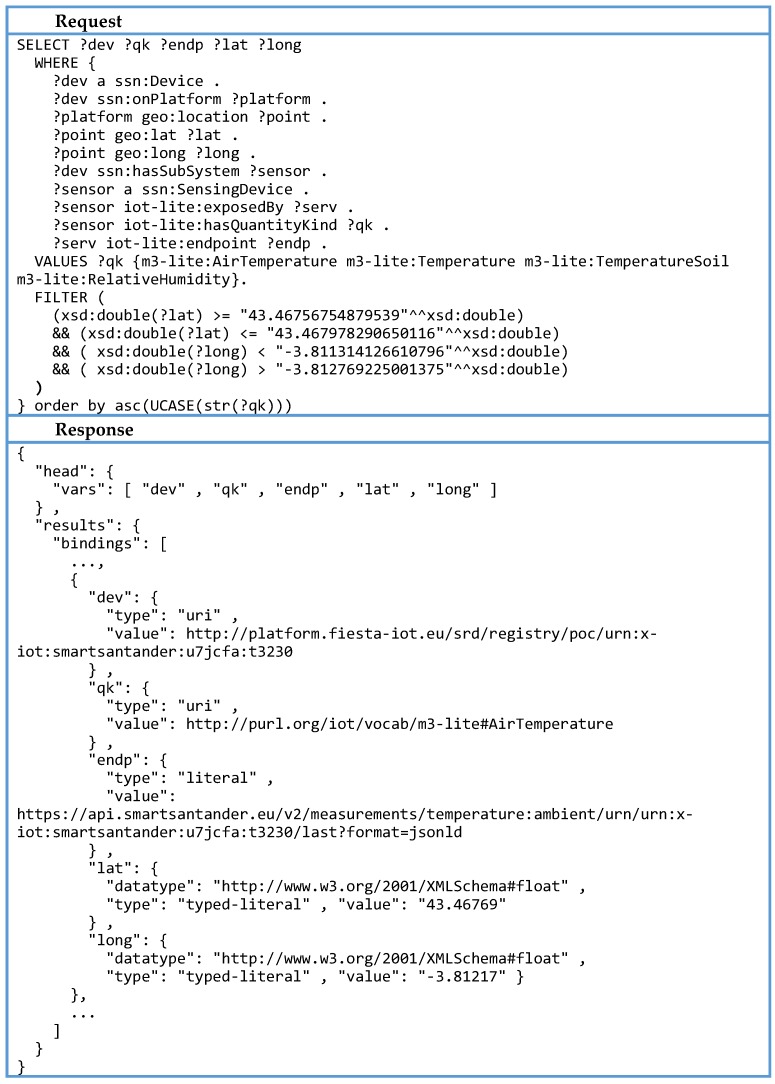
Example query/response for IoT Resource discovery.

In the PoC experimenter’s frontend, a request is launched once the desired phenomena is checked and the covered geographical space of the visualized map is highlighted. At the backend, a SPARQL query is created out of this information. First the selected human-readable phenomena descriptions are converted to the FIESTA-IoT taxonomy, which later on will be included in the SPARQL request. For instance, the temp tag used in the web interface compiles various M3-Lite entities related with temperature, as m3-lite:AirTemperature, m3-lite:Temperature or m3-lite:TemperatureSoil. This is the list included in the SPARQL request, as can be seen in Figure 13. Note that this GUI is taking the role of an experiment and each experimenter will be able to perform its own filtering mechanisms. Then, the query is finalized by the inclusion of the geographical boundaries as an additional filter.

Once the query is complete, it is sent to the SRD, whose semantic engine will construct the answer based on the available information. The response contains all the sensors that match the criteria. Every resource has a geographical position and an endpoint. Based on this geographical data a pre-result is sent to the GUI in order to show the resources located on the map. The list of endpoints is used to trigger a set of requests to receive the latest observation of the selected sensors.

**Figure 14:**
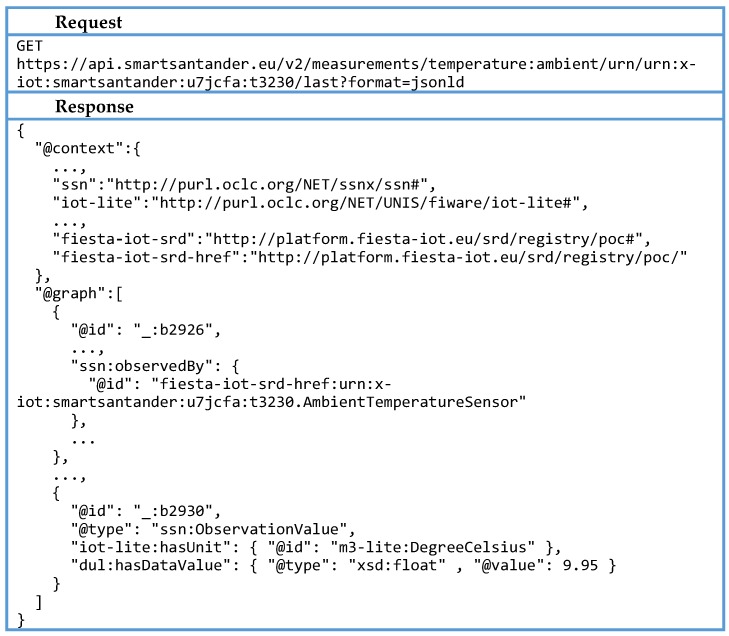
Example request/response for data acquisition.

For every resource gathered, an HTTP GET request to its IoT Service endpoints is sent. As can be seen in Figure 14, the response follows the FIESTA-IoT semantic description. In this case, the serialization MIME format requested was JSON-LD. The information displayed on the GUI is the type and value of the observation, as well as its timestamp.

**Figure 15:**
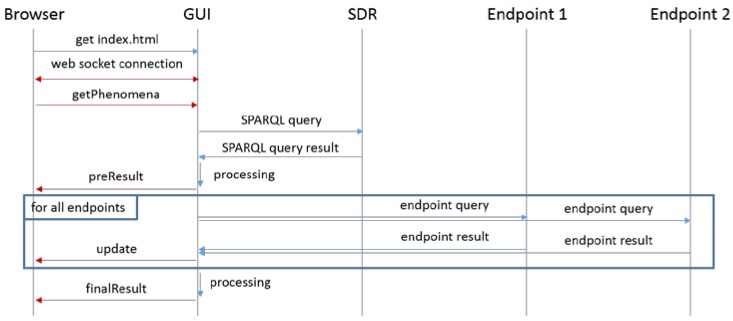
Sequence diagram of the process for retrieving Observations bound to phenomena.

For every received response, an update to the GUI is sent, and the map is filled with markers showing the node/observation position, resource type, observation quantity kind, value and unit of measurement. The process depicted above for accessing the observations available for a specific phenomenon is summarized in the sequence diagram shown in Figure 15, where the interactions and the exchange of requests/responses between all the involved components are shown.

**Figure 16:**
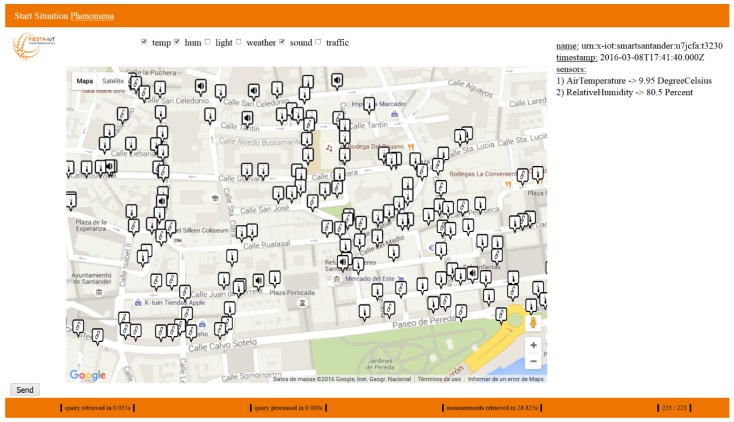
Screenshot of the Federation Resource Browser for a completed request.

The screenshot in Figure 16 graphically shows the result of a request for temperature, humidity and noise Observations within the specified area. The interface can be divided in 4 zones. On the top it is possible to select the kind of information requested (for the PoC only six phenomena were selectable but using a quantity kind taxonomy the selection could be extended to a drop list, for example). The center of the page presents a map with all the devices deployed in that area. Within the map it is possible to select any of the devices to get its information. When a device is selected, on the right hand side, the information it has observed appears. In the example, the markers in the map are showing the location of the resources, while the text on the right side shows the information bound to the selected one: its name, the timestamp, and the observations from the containing sensors.

At the bottom of the website, additional information can be seen. From left to right, we find the retrieving time of the SPARQL query to the SRD, the query processing time and the time needed to query all endpoints. The last entry is the number of sensors available and a counter with the number of received responses. If both numbers are equal, all endpoints are queried and after the processing the frontend will retrieve the final result.

From the results shown in Figure 16, which correspond to having retrieved the 223 different devices within the map boundaries, it is evident that retrieval and processing of information times (55 and 3 milliseconds respectively) are negligible. The measurements retrieval time, although reduced (0.13 s per resource), is considerable. However, this time is heavily dependent on the way the application has been implemented. The application made the endpoint queries (cf. Figure 15) in a sequential manner that was not optimal. Since the application is playing the role of the experiment, it is the experimenter responsibility to properly implement its own software to best exploit the meta-platform services. Of course, these results are not enough to lead to any remarkable conclusion on the system performance but, at least from an empirical point of view, they demonstrate that it can provide a more than acceptable quality of experience.

### 5.2. Smart City Performance Model Experiment

As second experiment we have envisioned a Smart City Performance Model. This is a platform which is capable of showing the status of a city leveraging data analytics algorithms. This FIESTA-IoT application has the objective to compute indicators about a city (or more generically about a geographic region). It summarizes the multiple situations related to the smart city domain (e.g., safety, environment etc.), but also the situations of the different IoT subsystems (e.g., quality of the IoT deployment, status of the IoT deployment etc.).

The indicators can be computed using different data analytics techniques like algorithms based on time-series, real-time analytics over the latest data observed, prediction of events, trend analysis, sensor fusion, etc. In this PoC we have focused our attention only on real-time analytics over the latest measurements in a geographic area. The indicators can be expressed in multiple different forms, like a discrete number (e.g., crowd level), a continuous number (e.g., average temperature), an array of values, a Boolean value, etc. In order to have a common understanding of the indicators’ outcome, we have chosen to use a traffic-light style color-code. It allows achieving a higher level of abstraction: red code if the situation expressed by the indicator is critical, yellow code if the situation needs the human attention or green code otherwise.

The indicators can be also visualized in the experiment map area. The area focused in the map is virtually divided in quadrants (the chosen value for this PoC is a 6 × 6 grid, thus 36 quadrants) and for each quadrant the real-time indicators are computed. In case of a resulting yellow or red code for the indicator, a yellow or red alert icon is drawn in the map and located in the corresponding quadrant. Zooming the map in or out or moving the focus of the map will cause a recalculation of the quadrants and then of the indicators. The indicators are computed taking into account the observations from the deployed sensors. In order to retrieve the needed data we have adopted the same approach described in Section 5.1.

As a PoC we have developed three indicators: two based on a single but abundant type of data (i.e., temperature measurements) which are computing respectively the *average* and the *peak*; and a third indicator describing *traffic* which is calculated using information from multiple traffic related sensors like road occupancy or traffic intensity The algorithms applied for each indicator are the following:

- For the *average* indicator, we have pre-processed the data and discarded outliers (assuming sensors malfunctioning). The ranges used for assigning the color code are dynamically computed calculating the quantiles of the dataset of the whole focused area. Then, for each quadrant, values are averaged and then checked against the computed ranges.
- For the *peak* indicator, we have not pre-processed the data since we are interested to know whether an abnormal temperature is measured (in this case the human attention is needed for checking if the situation is critical, e.g., a fire break, or if a faulty sensor needs replacement). Then, we have defined three fixed ranges of values for assigning the traffic-light color code. These ranges are empirically chosen accordingly to [42].
- For the *traffic* indicator, we have firstly averaged each type of data for each quadrant and then applied an empirically defined linear equation over the two different types. Then for each quadrant, we have applied a linear formula over the road occupancy average and traffic intensity average.

**Figure 17:**
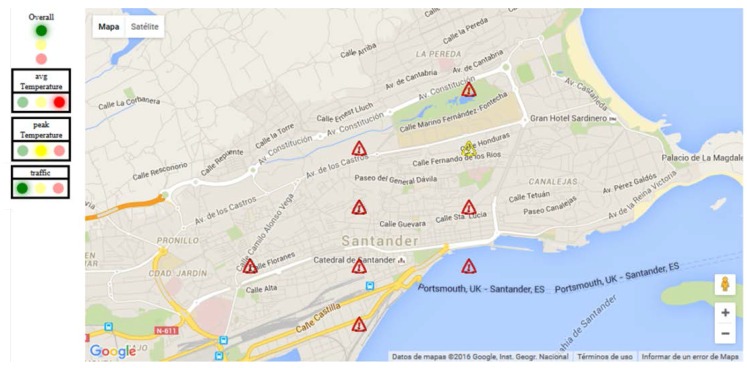
Smart City situation visualization map.

Figure 17 shows the user interface of the smart city platform centered to the city center of Santander. The outcome of the *average temperature* indicator, 36 values for all the quadrants, is depicted on the map. Only critical and warning situations are shown respectively through red and yellow alert icons.

The indicators related to each quadrant are further aggregated in order to show the status of the entire geographic scope shown in the map. The aggregation is made by checking the number of red and yellow flags over the total. The outcome of this aggregation is three flags related to the phenomena and traffic in the area: *average temperature status*, *peak temperature status* and *traffic status*. The three horizontal traffic lights on the left side of Figure 17 show those flags. The *average temperature* status is in a critical situation, the *peak temperature* is in a warning situation whilst the *traffic status* is normal.

Finally, a global aggregation has been made over the three situation flags in order to get the *global situation* indicator. The latter is represented by the vertical traffic light at the top left of Figure 17.

The Smart City Performance Model has two main requirements: the portability of the experiments and the access to multiple data sources in an agnostic way. The first requirement is meant for an automatic configuration of the experiment in terms of geographic scope. The data is requested and retrieved dynamically every time the scope of the map is shifted or zoomed. In our particular case, when the scope of the map was pointing to the Santander city, the data used was the one from the SmartSantander deployment; when the map was pointing to Guildford city, the data used was the one produced by the SmartCampus of the University of Surrey. Thanks to the semantic discovery and the retrieval of the semantically annotated data, we could reuse our smart city platform seamlessly without the need to implement more than one data parser. The reason behind the second requirement is to leverage the dataset from multiple IoT system located in the same geographic area. This will speed up the integration of new IoT deployment in our Smart City Performance Model. In case of this PoC, we have experienced the fulfilling of this requirement by calculating the situation of Western Europe.

## 6. Conclusions

This paper has presented the specification and implementation of an IoT experimentation meta-platform resulting from the federation of two different testbeds. The two testbeds have been briefly described to let the reader grasp its main differences and assess the challenges under its federation. The overall EaaS concept pursued is realized through the creation of a meta-platform that virtualizes the underlying testbeds. The available resources and data (i.e., observations) are exposed through homogeneous interfaces that hide internal transformation and management. The approach followed for the design and implementation of the meta-platform described in this paper is the use of semantic technologies for guaranteeing interoperability among heterogeneous IoT platforms. The ontology that is at the core of these semantic technologies has been sketched (its detailed description is out of the scope of this paper). However, a detailed description of how this ontology has been used for the mapping of resources and observations from the federated testbeds has been provided. Last but not least, the article has presented two applications that have been implemented to take the role of experiments over the resulting meta-platform. This way, the paper has shown how the circle is closed and, most importantly, has demonstrated through a real-world use case (i.e., smart city situation analysis service), the potentialities of working with a semantically-enabled federation of IoT testbeds.

The system that we have implemented has a twofold objective. On the one hand, it serves as a proof-of-concept implementation meant to create awareness about the future meta-platform and its experimentation support capacities. On the other hand, it is the baseline on top of which future components will be integrated to create the full-blown EaaS platform. In this sense, next steps will focus on completing the specification and implementation of the FIESTA-IoT platform. Moreover, besides the integration of functionalities, more testbeds will be federated, thus, enlarging the quantity and diversity of datasets and data-streams available for consumption by the experimenters.

Finally, since the focus of the article is to present the proof-of-concept implementation that the authors have carried out, performance evaluation has not been considered. As the implementation is missing some functionalities that will only be available in the first release of the FIESTA-IoT platform (the one that will actually be opened for experimentation), this kind of performance results will be subject of future work. Anyhow, some performance details of the integrated system have been included in the Federation Resource Browser application and discussed accordingly as they are not completely representative of the platform but depends also on the application itself.
